# The Central Role of AMP-Kinase and Energy Homeostasis Impairment in Alzheimer’s Disease: A Multifactor Network Analysis

**DOI:** 10.1371/journal.pone.0078919

**Published:** 2013-11-12

**Authors:** Laura Caberlotto, Mario Lauria, Thanh-Phuong Nguyen, Marco Scotti

**Affiliations:** The Microsoft Research - University of Trento Centre for Computational Systems Biology, Rovereto, Italy; Semmelweis University, Hungary

## Abstract

Alzheimer’s disease is the most common cause of dementia worldwide, affecting the elderly population. It is characterized by the hallmark pathology of amyloid-β deposition, neurofibrillary tangle formation, and extensive neuronal degeneration in the brain. Wealth of data related to Alzheimer’s disease has been generated to date, nevertheless, the molecular mechanism underlying the etiology and pathophysiology of the disease is still unknown. Here we described a method for the combined analysis of multiple types of genome-wide data aimed at revealing convergent evidence interest that would not be captured by a standard molecular approach. Lists of Alzheimer-related genes (*seed genes*) were obtained from different sets of data on gene expression, SNPs, and molecular targets of drugs. Network analysis was applied for identifying the regions of the human protein-protein interaction network showing a significant enrichment in *seed genes*, and ultimately, in genes associated to Alzheimer’s disease, due to the cumulative effect of different combinations of the starting data sets. The functional properties of these enriched modules were characterized, effectively considering the role of both Alzheimer-related *seed genes* and genes that closely interact with them. This approach allowed us to present evidence in favor of one of the competing theories about AD underlying processes, specifically evidence supporting a predominant role of metabolism-associated biological process terms, including autophagy, insulin and fatty acid metabolic processes in Alzheimer, with a focus on AMP-activated protein kinase. This central regulator of cellular energy homeostasis regulates a series of brain functions altered in Alzheimer’s disease and could link genetic perturbation with neuronal transmission and energy regulation, representing a potential candidate to be targeted by therapy.

## Introduction

Alzheimer’s disease (AD) is a neurodegenerative disorder characterized neuropathologically by the extracellular accumulation of amyloid-beta plaques and the intracellular accumulation of hyperphosphorylated tau protein, the neurofibrillary tangles [1]. AD is the most prevalent neurodegenerative disorder worldwide and it is a complex disease associated with multiple genes [2]. Although a large body of literature focuses on the importance of a few key proteins for AD onset and progression, our understanding of the etiopathology of the disease is still very limited. Current medical treatments for AD are purely symptomatic and hardly effective [3], thus, the understanding of the molecular mechanisms underlying AD is essential for the development of novel therapies.

Over the last decade, many studies have been devoted to dissecting the molecular pathways involved in AD using a variety of experimental designs and technological approaches, including genomic-wide linkage scans [4], genetic association studies [5], and microarray gene expression investigations [6]–[11]. In the present study, a systems biology approach was applied to extract overlapping evidence from different sources of AD-related data. Our convergent analysis of different data types enabled us to overcome the limitation of analyzing each single data type in isolation and to provide a multi-source, unbiased view of the evidence embedded in the genomic, transcriptomic, and drug molecular targets. As a final step, Alzheimer’s disease associated genes and genetic phenotypes collected in the Online Mendelian Inheritance in Man (OMIM) database representing the consolidated knowledge on AD were integrated in the analysis to validate the method. Previous computational studies have tried to integrate different text mining approaches, genetic, functional or *-*omics data to provide hypotheses for the biological mechanisms underlying the pathology [12]–[15]. This is the first attempt to integrate the genomic aspect of AD with the gene expression and drug candidate targets. We have used AD-related data obtained from multiple sources: (1) transcriptomic data of six different post mortem brain regions of AD affected subjects [11], analyzed using a newly developed analytical method [16], (2) single nucleotide polymorphism (SNP) data integrated from multiple studies [17], (3) molecular targets of Alzheimer’s drugs in the different phases of the drug discovery process, and, for the validation step, (4) genes associated to Alzheimer’s disease extracted from the Online Mendelian Inheritance in Man (OMIM) database [18]. These sets of data were used to derive lists of *seed genes* and represented the basis to perform network analysis. We then used a protein-protein interaction (PPI) network as a scaffold on which to embed the lists of *seed genes*, with the lists considered both separately and in different combinations.

**Figure 1:**
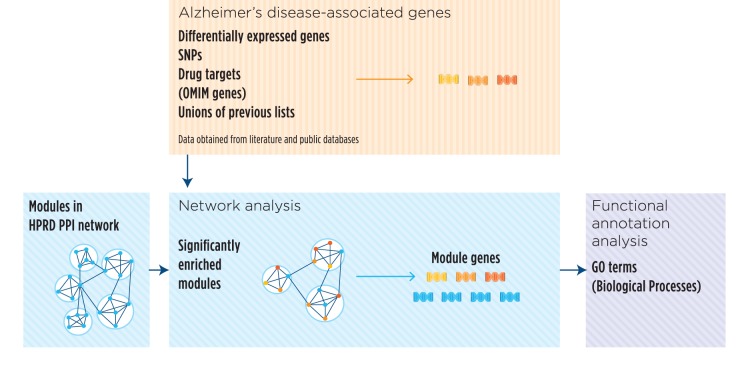
Schematic representation of the network analysis workflow. Significant gene expression signatures associated to AD were extracted from the GSE5281 dataset, while lists of SNPs, drug targets, and OMIM Alzheimer’s genes were obtained from public databases. These *seed genes* (in yellow and orange) inform about transcriptomic and genetic properties of AD, also providing details on drug targets in the different phases of the drug discovery process and AD associated genes in the OMIM database. Following module structure detection in the protein-protein interaction (PPI) network derived from HPRD data (see groups of nodes in the same white-background circles), we investigated the presence of reference modules where *seed genes* (obtained through the three simple lists and their union) were over-represented. We characterized the functionality of these enriched modules by testing over-represented Gene Ontology biological process terms, both considering *seed genes* (in yellow and orange) and non-*seed genes* (in light blue) that closely interact with them.

A number of methods have been proposed for integrating experimental data and prior knowledge in the form of PPI interactions. Some of the existing tools implement network building methods whose starting point is a list of genes, which are then used as a backbone for the iterative assembling of connected networks [19]. Others, such as in Komurov et al. [20], start considering the whole network structure and then proceed to assign weighs to nodes to reflect the levels of gene expression from microarray data. In the present paper, we have developed an intermediate approach. We have used the whole interaction network from HPRD [21], partitioned it into modules and tested their enrichment in terms of *seed genes*. We have, then, characterized the biological properties of the significantly enriched reference modules by studying the over-represented GO biological process (GOBP) terms (Figure 1). Our method combines the merits of the holistic perspective considering the whole network structure, allowing the concurrent comparison of different data types.

This biomolecular network has provided a richer setting to characterize genes found to be involved in AD and to identify AMP-activated protein kinase (AMPK) signaling, a metabolic sensoring pathway and energy regulators including neuropeptides, as a major player in the pathophysiology of AD, which could explain various aspects of AD pathogenesis.

## Materials and Methods

### *Seed Genes* Lists

The lists of *seed genes* (1) extracted from gene expression data, (2) identified with significant SNPs, (3) obtained after data search for drug targets, and (4) retrieved from OMIM database were obtained as described in the following, and are reported in Tables S1.

#### Gene expression *seed genes*

Microarray data were downloaded from Gene Expression Omnibus (GEO; http://www.ncbi.nlm.nih.gov/geo/). Dataset GSE5281 [11] refers to a series of brain regions differentially affected by Alzheimer’s disease and was selected based on the good quality of the experimental design. Full description of the dataset is reported in [11]; briefly, histologically non-affected neurons were collected by laser-capture microdissection from six different brain regions: entorhinal cortex (EC), hippocampus (HIP), medial temporal gyrus (MTG), posterior cingulate cortex (PC), visual cortex (VCX), and superior frontal gyrus (SFG). The study population consisted of 11–13 elderly controls and 10–23 AD affected subjects for each region. The pre-processed version of dataset GSE5281 was downloaded and used without modifications. In order to derive lists of relevant genes, we first obtained AD differential expression profiles by dividing each AD profile by the average of the controls for the respective region (i.e., the HIP profiles in AD patients by the average of HIP profiles in controls). We then ranked each profile separately, from the most expressed to the least expressed probeset; at the end of this step, each probeset had a separate rank assigned to it for each of the expression profiles. In order to obtain a brain region-specific ranked probeset list, we summed the ranks for each region separately, and then we re-ranked the probesets according to the rank sums. Finally, the top 125 and the bottom 125 probesets were collected for each region, to form a brain region-specific list. The value of the length of these lists (125+125) was selected as the one that gave the best partitioning of the map of samples in well-defined groups, thus corresponding to a maximally informative and minimally redundant expression signature. We have shown that the signature length is not critical, in the sense that usually the range of values resulting in a satisfactory clustering of the map is quite wide [16], [22].

The map was obtained by measuring the reciprocal distance between the lists extracted from each profile, and then representing such distances in the form of a graph (Figure S1), as detailed elsewhere [16].

#### SNPs *seed genes*

SNPs data were obtained from the AlzGene database (www.alzgene.org). Only highly significant meta-analysis results (p-values <0.00001) were used to select a subset of SNPs – AD-associated genes confirmed by numerous studies [17]. We tested separately the complete dataset (533 SNPs) as well for the additional statistical analysis.

#### Drug targets *seed genes*

Drug molecular targets were obtained by collecting information from different pharmaceutical company websites and from a clinical trial database (www.clinicaltrials.gov). Drugs in all phases of the drug discovery process, from preclinical to marketed drugs, were included. This allowed obtaining the broadest coverage of the genes of interest for pharmaceutical drug development to identify the overall key molecular targets of interest for the treatment of AD. Only primary targets were considered as *seed genes* for network analysis.

#### OMIM *seed genes*

Alzheimer’s disease genes and genetic phenotypes were extracted from the OMIM database, using Alzheimer’s disease as reference keyword [18].

### Network Construction and Analysis

For protein interaction data, we used the 2009 version of the Human Protein Reference Database (HPRD; http://www.hprd.org/). This is a literature-curated human PPI interaction network comprising 37039 interactions among 9617 genes [21]. Nodes of the network are the genes (named with gene symbols), while edges stand for protein-protein interactions (e.g., enzymatic, regulatory, transcriptional). We removed loops (edges for which the two endpoints are the same gene) and duplicate edges (interactions with the same two nodes that are listed more than once), and identified the maximal connected component of the network (giant component). The final network was composed of 9219 genes and 36900 interactions. To find network modules, we analyzed the final network with the “spinglass.community” function [23] included in the R package igraph [24]. Network modules represent cohesive subgroups composed of genes that are more intimately interconnected among each other than with the rest of the network. Using the “spinglass.community” function, these modules are identified only considering the arrangements of network interactions. Since the module detection function maximizes the modularity by adopting a heuristic approach, module structure (i.e., number, size and node composition) might slightly change in different runs [23], [25]. To deal with this, we ran the “spinglass.community” partitioning algorithm 100 times. At each run, we performed hypergeometric tests (p-values threshold 0.05) using the “multiHyperGeoTest” function from the R package HTSanalyzeR [26] to identify the network modules that were significantly enriched with *seed genes* considered for that run (differentially expressed genes, SNPs, drug targets, OMIM genes or lists obtained from their union). When more enriched modules were found, we compared their size and composition to identify the substantially overlapping modules across multiple runs (Figure S2). First, we grouped enriched modules based on their size, applying the function “hist” of the R package graphics (and using the option: breaks = "Sturges"; see [27]). The composition of significant modules of similar size was compared and eventually merged into a new reference module summarizing the results of multiple runs (i.e., in case this did not alter significant statistics on gene enrichment). Each reference module was obtained by selecting the largest number of genes and interactions found with different runs. To avoid excluding genes and interactions of possible interest, we considered the largest amount of genes and interactions as representative of each reference module, when this did not compromise the statistics for over-represented *seed genes*. If the significant enrichment with *seed genes* vanished after the union of more modules, they were considered as representative of different communities and analyzed as separate sub-networks. The highest variability was observed for the changes in the number of interactions while module composition was more stable during different runs. We also required all reference modules to be constituted by sets of connected nodes. Once the composition of these reference modules was identified, we used the whole lists of “reference module genes” to extract the most representative GO biological process terms (i.e., the ones that are over-represented, but that do not refer to most general biological processes). For identifying and visualizing enriched GO terms, we used GOrilla and REVIGO tools; hypergeometric distribution was applied to test GO term enrichment, and a p-value threshold of 0.001 was selected [28], [29].

### Statistical Analyses on the Relatedness of AMPK to AD

In order to characterize the relevance of AMPK system in AD a series of three types of statistical analysis was performed. AMPK is represented in the final network by 9 nodes: 2 protein kinase, AMP-activated, catalytic subunits (i.e., PRKAA1, PRKAA2); 5 protein kinase, AMP-activated, non-catalytic subunits (i.e, PRKAB1, PRKAB2, PRKAG1, PRKAG2, PRKAG3), the acetyl-CoA carboxylase alpha (ACACA) and beta (ACACB). They result in a sub-network of 9 nodes collectively called “AMPK nodes”. These nodes are surrounded, in the final network, by 25 direct neighbors. Altogether, they result in a sub-network of 34 “AMPK nodes+neighbors”.

First, we measured the frequency of the 9 AMPK nodes and 34 “AMPK nodes+neighbors” in the enriched modules. We applied the Shapiro-Wilk test (function “shapiro.test” from the R package nortest) to assess the normality of the count distributions for AMPK and non-AMPK nodes. We used the Wilcoxon rank sum test (function “wilcox.exact” from the R package exactRankTests) to investigate whether, in reference modules, AMPK nodes were characterized by significantly higher frequencies than non-AMPK nodes.

Second, we checked whether the sub-network of 34 “AMPK nodes+neighbors” was significantly enriched with *seed genes* of different origin, considering first the lists of *seed genes* separately and, then, their union. Enrichment was evaluated with the hypergeometric test and p-values were adjusted with the Benjamini and Hochberg correction [30].

Third, we measured average and global patterns of shortest distances linking the 34 “AMPK nodes and neighbors” to *seed genes*, and compared their distributions to 1000 subsets obtained by randomly sampling 34 non-*seed* and non-AMPK nodes from the final network using the Wilcoxon signed rank test, following evaluation of normality of the distributions with the Shapiro-Wilk test. Comparisons were carried out using both 34 average shortest paths (“avg” scenario) and considering the whole distribution of shortest paths to *seed genes* (“all” scenario). For each comparison, we combined the 1000 p-values into a unique p-value by considering that p-values should be uniformly distributed when the null hypothesis is true (i.e., when there are no differences between the distributions of shortest paths obtained with AMPK and non-AMPK nodes) [31].

In presence of *n* random uniform variables, the cumulative distribution function is as follows:
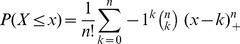
(1)


The left-hand side stands for the probability that the random variable *X* takes on a value less than or equal to *x*; given the *k*-th element, the expression *(x – k)* indicates the positive part of *(x – k)*: it equals *(x – k)* if *(x – k)* is positive and equals 0 otherwise. Due to the central limit theorem [32], as the number of random uniforms increases, the sum will converge to a normal distribution with mean *n*/2 and variance *n*/12. This convergence can be used to estimate a combined p-value for each set of 1000 p-values. If the p-values are lower than expected from the null, the sum would show up in the lower tail of the distribution.

## Results

**Figure 2:**
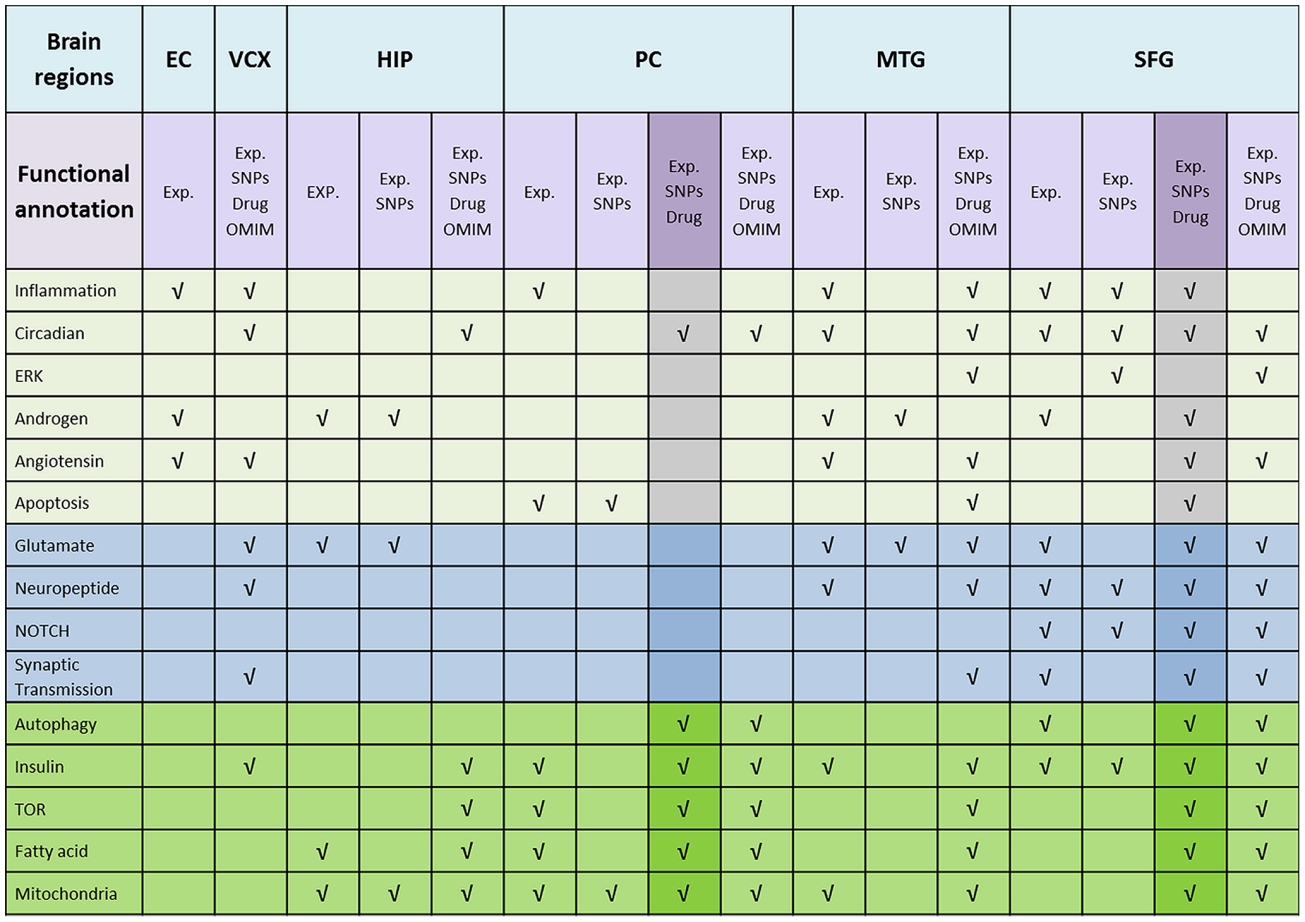
Summary of statistically significant Gene Ontology biological processes functional annotation corresponding to genes in enriched reference modules. Data refers to reference modules obtained using gene expression only (expression), by integrating this information with SNPs (expression+SNPs), and by merging the mRNA expression data, SNPs and drug targets (expression+SNPs+drug targets), and by combining the mRNA expression data, SNPs, drug targets, and OMIM genes (expression +SNPs+drug targets+ OMIM). In light blue are GO terms associated to synaptic transmission and neuronal signaling, in dark green are metabolism-associated GO terms, in gray remaining relevant terms. Highlighted are the results which have been discussed in detail in the discussion section. Specific GO terms are described in Table S2.

We carried out functional enrichment analyses of GO biological process terms for the reference modules representing unique network communities characterized by over-represented *seed genes*. These modules were determined for each *seed genes* list separately (i.e., gene expression, SNPs, drug targets, and OMIM genes) and then by considering (1) the union of gene expression and SNPs, (2) the union of gene expression, SNPs, and drug targets, and (3) the union of gene expression, SNPs, drug targets, and OMIM genes (Figure 2). After analyzing gene expression data, several reference modules were found for all brain regions and their number ranged across the regions, with SFG having the highest number of reference modules and VCX the lowest. An enriched reference module was also found associated to drug targets, numerous modules to OMIM genes associated to Alzheimer’s disease, while none linked to SNPs (Tables S1). The number of genes per reference module ranged from 13 to 1885.

Integrating the most significant SNPs with expression data, enriched reference modules were identified only for four brain regions: HIP, PC, SFG, and MTG. Adding drug targets to the analysis, we found enriched communities only in three brain regions: PC, MTG and SFG, while including in the analysis OMIM genes five brain regions (PC, MTG, HIP, VCX, and SFG) were associated to enriched modules (Figure 2).

Complete lists of genes in the enriched modules are summarized in Tables S1.

**Figure 3:**
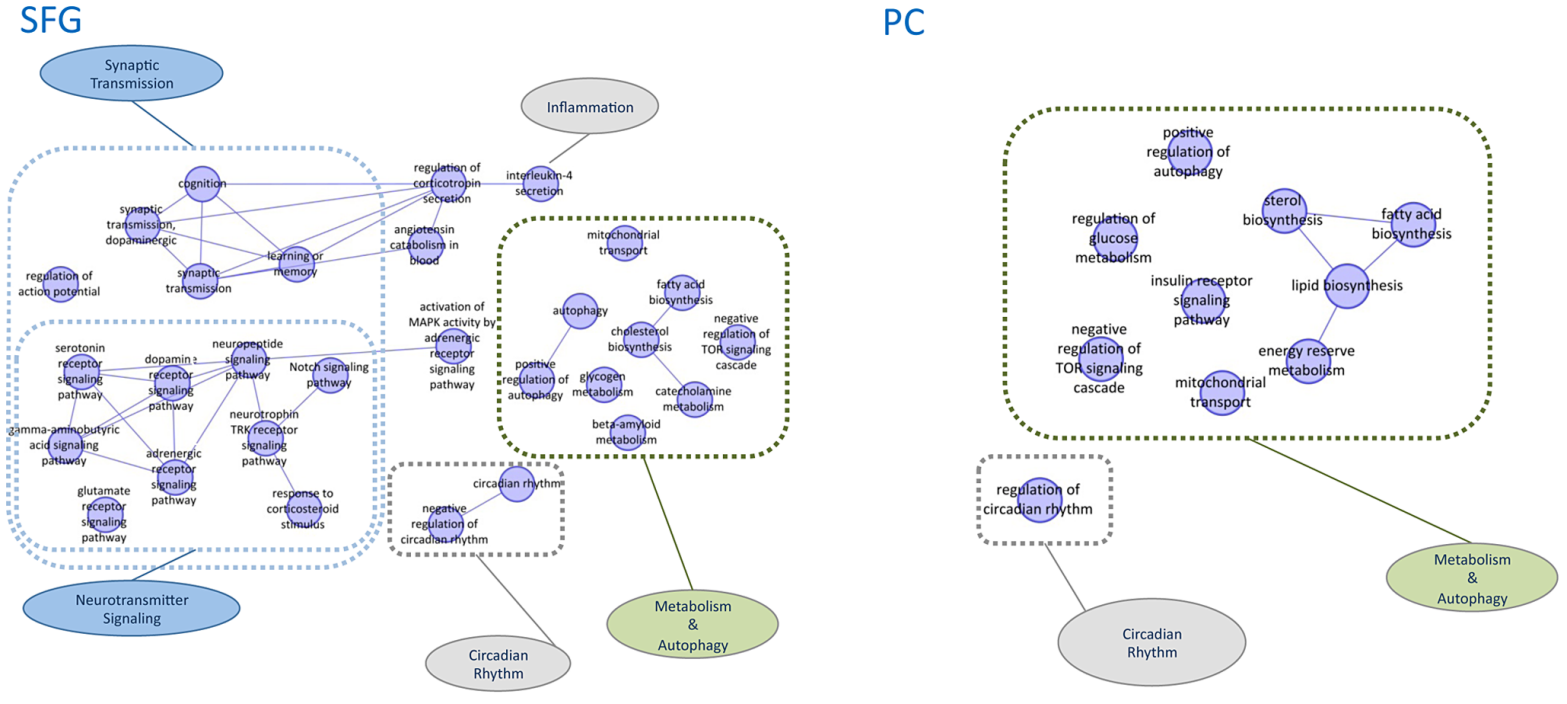
Schematic graphs of over-represented Gene Ontology biological process terms in enriched SFG and PC reference modules. *Seed genes* were obtained from the union of differentially expressed genes with most significant SNPs and primary drug targets. GO terms are represented as nodes, and the strongest GO term pairwise similarities are designated as edges in the graph. GO terms are grouped to illustrate the main metabolic signature in PC, while both metabolic and synaptic transmission functions characterize SFG.

Table 1 and Figures 2–3 describe the functional annotation analysis results. Overall, reference modules related to expression data only in HIP and PC cortical area were mainly related to metabolism, while in neocortical regions such as MTG and SFG, both metabolic and higher brain biological process terms related to neuronal transmission (e.g., neuropeptide, NOTCH, and synaptic transmission) were represented. Integrating the SNPs and drug targets data to the expression analysis, PC maintained the “metabolic”-related profile, while in SFG, beside the fact that the neuronal transmission biological function annotation was retained, additional GO terms associated with metabolism were included (Figures 2–3). Including OMIM data in the analysis, a role for circadian rhythm was evident in the five brain regions (PC, HIP, SFG, MTG, and VCX). A metabolic profile was still associated to PC and HIP, SFG and MTG were related to both metabolic and higher brain functions activities, while VCX was associated to synaptic transmission. The complete list of specific GO terms can be found in Table S2.

**Table 1:** Gene lists associated to main classes of Gene Ontology biological process terms.

Fatty acid		Mitochondria			TOR		Autophagy			Insulin		Circadian	
SFG Exp.SNPs Drug	PC Exp.SNPs Drug	SFG Exp.SNPs Drug	PC Exp.SNPs Drug		SFG Exp.SNPs Drug	PC Exp.SNPs Drug	SFG Exp.SNPs Drug		PC Exp.SNPs Drug	SFG Exp.SNPs Drug	PC Exp.SNPs Drug	SFG Exp.SNPs Drug	PC Exp.SNPs Drug
ACACA	ACACA	**PRKAG2**	PRKAG3	PRKAA1		PRKAA1		ATG4A	PRKAA1	PFG	PRKAG1	CRH	PRKAA1
**ACACB**	**ACACB**	PRKAB2	PRKAB3	PRKAA2		PRKAA2		ATG10	PRKAA2	PDX1	**PRKAG2**	ADA	PRKAA2
PRKAA1	PRKAA1	**ACAB**	**ACAB**					ATG7		FFAR1	PRKAB1	**ADORA1**	
PRKAA2	PRKAA2	PRKAA2	PRKAA3					MAP1LC3B		ANXA1	PRKAB2	GHRL	
PRKAB1	PRKAB1							ATG5		NEUROD1	PRKAA1	**DRD2**	
PRKAB2	PRKAB2							GABARAP		MC4R	PRKAG3		
PRKAG1	PRKAG1							NBR1		CAMK2G	PRKAA2		
**PRKAG2**	**PRKAG2**							ATG4B		FKBP1B			
PRKAG3	PRKAG3							GABARAPL2		MAFA			
										CACNA1C			

Comparative gene lists associated to main classes of Gene Ontology biological process terms derived by integrating gene expression, SNPs and drug targets data in SFG and PC. In few cases (Fatty acid and TOR signaling) the gene list are perfectly matching, while in Insulin, Autophagy and Circadian Rhythm, they differed considerably. *Seed genes* are in bold.

PRKAA1-2, PRKAB1-2 and PRKAG1-3 are AMPK subunits, while ACACA and ACACB are ACC.

Comparing the gene lists associated to the groups of similar GO terms among different brain regions and using different subsets of data, in most cases there was a good overlap (e.g., Fatty acid and TOR), while in a few other cases they resulted very different (Table 1).

Through GO enrichment analysis, we found that AMP-kinase signaling pathway plays a central role in AD. To further corroborate this outcome, we tested the statistical relevance of AMPK-related nodes to AD. We observed that the frequency of both 9 AMPK nodes and 34 AMPK-related nodes was significantly higher than the frequency of non-AMPK nodes, in case of enriched reference modules obtained from the union of gene expression, SNPs and drug targets. The sub-network of 9 AMPK nodes and their 25 direct neighbors was significantly enriched with different types of *seed genes*, especially when considering the extended list of 533 SNPs. In general, SNPs were significantly over-represented in the surrounding of AMPK. The 34 AMPK-related nodes showed shorter distances to whole SNPs, drug targets and OMIM genes in HPRD, if compared to other non-AMPK and non-*seed genes*. Results on the AMPK relatedness to AD are summarized in the Table S3.

We have then investigated whether the reference modules identified with lists of *seed genes,* obtained using gene expression profiles, SNPs, and drug targets, were significantly enriched with OMIM genes (p-values were estimated with hypergeometric tests - see the Benjamini & Hochberg correction; adjusted p-value threshold = 0.1). Results confirmed the outstanding importance of SFG (altogether, 11 OMIM genes out of the 13 found in HPRD were included):

3 SFG modules (expression data only) were enriched with OMIM genes (i.e., these 9 genes: PSEN2, BLMH, PSEN1, PLAU, APOE, APP, HFE, MPO, A2M).
2 SFG modules (expression data & SNPs) were enriched with OMIM genes (i.e., these 8 genes: PSEN2, PSEN1, PLAU, APOE, APP, HFE, MPO, A2M).
2 SFG modules (expression, SNPs & drug targets) were enriched with OMIM genes (i.e., these 5 genes: PSEN2, BLMH, PSEN1, NOS3, ACE).

## Discussion

The novelty of our investigation is in the approach we used in integrating multiple data types in order to elucidate the etiopathology of AD. Our approach can be described as follows. We sought to combine three different types of data specifically selected for their potential to shed light on the molecular details of AD: transcriptomic data in the form of expression profiles in brain, genetic data in the form of SNPs, and affected pathways in the form of drug targets.

The starting point of our analysis was a PPI network (data extracted from the HPRD dataset), which we used as a scaffold to merge the information derived from the three sets of data. We applied network analysis for extracting the hints on AD-specific mechanisms contributed by these three datasets and for revealing possible overlaps in the biological process terms they refer to. A preliminary module analysis of the PPI network was performed, a module being a group of nodes (proteins) characterized by a higher degree of connectivity to other members of the group than to non-group nodes, assuming that genes with a highest number of structural connections are also better candidates for more intense patterns of functional interactions. The aim was finding AD-pertinent enriched modules (i.e., modules showing a significant over-enrichment of AD-related genes) in the human PPI interaction network, for characterizing the most relevant biological processes associated to these reference modules. We introduced a novel approach for estimating reference module composition applying a heuristic algorithm for the concurrent analysis of heterogeneous experimental data [23]. We avoided overweighting the importance of a specific data type and, given this choice, we were unable to utilize an exact method which requires the integration of network structure with additional properties concerning nodes and edges, an otherwise excellent solution in case of mono-dimensional experimental data [20], [33]. Other studies consider the complete network structure for identifying disease genes [34] or performing functional analyses of genomic data [20]. However, the most prevalent software tools (e.g., GeneGO and Ingenuity Pathway Analysis) adopt list-based network building methods (i.e., they construct *ad-hoc* networks through an iterative process, by including neighbors of *seed genes* up to a given distance), or score pre-defined pathways and functional terms that are over-represented by lists of *seed genes* [35], [36]. Since our approach combines holistic view (i.e., it uses the whole network structure) and module detection of an unweighted network (i.e., it estimates module composition with a heuristic algorithm, by ranking at the same level all of the experimental data types) we argue that it is especially suitable for integrating multiple data types.

The additive role of the data types can be best appreciated by looking at the significance analysis of AMPK for one, two, three or four datasets. Table S3.2 (in Table S3) shows that none of the four data sets is by itself sufficient to identify AMPK, and instead the use of all three supporting sets (transcriptional, SNP, drug targets) is necessary for its identification. The addition of OMIM, which represents the consolidated knowledge on AD and does not include AMPK (Table S3.1, in Table S3), has the effect of diluting the supporting evidence for new genes in favor of established ones, and brings the significance of AMPK below threshold.

The functional properties of the areas of the network enriched in terms of the three sets of AD-genes (expression, SNPs, and drug targets) were characterized and revealed that, in posterior cingulate cortex, the metabolism-related terms display greatest importance, with particular relevance of insulin, fatty acids and mitochondrial functions (Figures 2–3). Posterior cingulate cortex is metabolically affected in the early phases of AD [37] and genes influencing mitochondrial energy metabolism were found to be down-regulated in AD patients [10]. However, the subset of genes identified by Liang and colleagues refers to a great proportion of the nuclear genes encoding mitochondrial ETC (electron transport chain) subunits in PC, including TIMMs and TOMMs, which are required for the transmembrane mitochrondrial transportation of ETC components, thus differing from the genes highlighted by our study (Table 1). The genes associated to metabolism-related GO terms (fatty acid, insulin, mitochondria, mTOR signaling) in PC have as common and central molecules different subunits of AMP-activated protein kinase (AMPK; PRKAA1-3, PRKAB1-3; PRKAG1-3) and AMPK enzyme complex ACC (ACACA, ACACB). AMPK is a cellular complex involved in intracellular energy metabolism, a regulator of energy homeostasis. Interestingly, analyzing the enriched modules of drug targets and gene expression data separately, the same genes were found, with a convergence to AMPK signaling using data of very different origin (Tables S1). This energy-sensing enzyme is linked to different molecular functions that are altered in AD such as defects in glucose uptake [38], mitochondrial dysfunctions [39] and alteration of autophagy pathways [40]. Recent studies suggest a role for AMPK in modulation of tau protein phosphorylation and amyloidogenesis, the major hallmarks of AD. Latest research indicated an upstream role for AMPK pathway as a critical mediator of the synaptotoxic effects of amyloid beta [41]. Thus, it is possible that the altered functionality of AMPK system in AD patients contributes to a neuronal imbalance in handling energy requirements, leading to higher Aβ and phospho-tau. AMPK is also involved in transmitting energy-dependent signals to the mammalian clock, thus regulating circadian rhythm; circadian rhythm disturbances have been well documented in AD as being part of the disease process, or a reflection of it [42]. The involvement of AMPK is further corroborated by previous transcriptome studies in AD post mortem brains where AMPK-related genes were found to be altered in prefrontal cortex of affected individuals, with a subunit-specific effect [7]. Also tacrine, an acethylcholinesterase inhibitor widely used for the treatment of AD, was shown to induce up-regulation of AMPK subunits in an in vitro model (E-MTAB-798 in expression ATLAS http://www.ebi.ac.uk/gxa/) [43]. Further evidence of the central role of AMPK in AD originates from preclinical and clinical studies. In an animal model of AD, the triple transgenic mouse model, pioglitazone treatment, an AMPK activator, results in the reduction of amyloid plaque, reduced inflammation and reversal of disease-related behavioral impairment [44]. In a recent clinical trial, rosiglitazone, an anti-diabetic drug acting on AMPK, was associated with improved cognition and memory in patients with mild to moderate AD [45].

**Figure 4:**
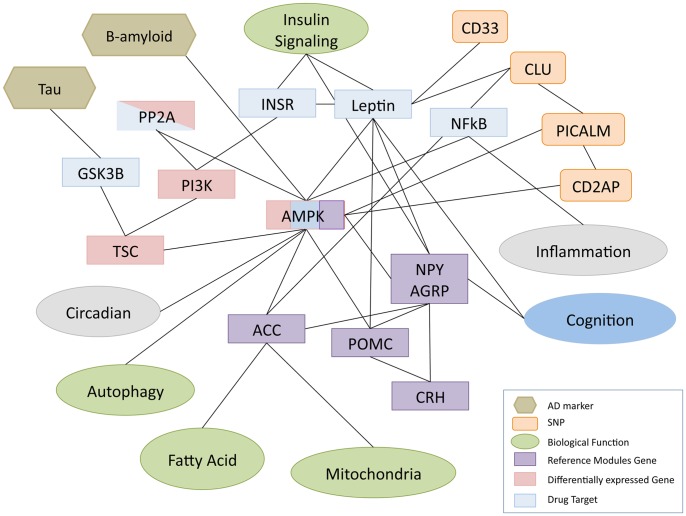
Simplified schematic graph of AMPK interactions with. (1) genes included in the enriched reference modules (purple), (2) differentially expressed genes (pink), (3) drug targets (light blue), and (4) SNPs (orange). Ellipses show the biological process terms associated to the genes (color as in Figure 2) and altered in AD; rhomboid shapes stand for histological markers of AD.

In order to associate AMPK functions to genetic alteration in AD, we investigated the molecular interactions between SNPs and AMPK-related genes found in the AD enriched modules. We found that three out of ten SNPs-associated genes in the lists of the most significant SNPs have a direct relation to AMPK: a genetic interaction for (1) CLU with ACC (ACACA) and (2) PICALM with AMPK (PRKAA1/PRKAG2) [46], and co-expression for (3) CD2AP with AMPK (PRKAB1) [47] (Figure 4). Also CD33, another gene characterized by a polymorphism that is significantly associated to AD, is related to AMPK, although indirectly, through leptin (Figure 4), another key player in energy regulation whose effects in inhibiting amyloid β production and tau phosphorylation are dependent on activation of AMPK [48]. Statistical analysis demonstrates also the closeness of AMPK-related genes to SNPs in comparison to other nodes in the network. This finding could provide evidence on the functional role of these loci in the mis-modulation of energy homeostasis, a scenario that assigns to energy impairment important roles in predisposing the brain to the etiology and pathogenesis of this condition.

The advocated role of AMPK and direct neighbor genes in AD was also supported by statistical analyses. Different lists of *seed genes* that are relevant for AD were overrepresented in the sub-network composed of AMPK genes and their direct neighbors. In addition, AMPK-related nodes showed significantly shorter distances to SNPs, drug targets and OMIM genes in comparison to randomly chosen nodes from the network.

Recently, a specific Alzheimer’s network was proposed by Mizuno and colleagues [49], a catalogue mapping of AD signaling pathway based on literature mining. Thus, we tried to merge this AD network with the enriched reference module (obtained from the integration of the three datasets). Among the few overlapping genes (ULK1, INPP5K, CIB1, PRKG1, SR1, ADRBK1, GNAQ, UBE2M, PCSK1, PRKAA2) an AMPK subunit, PRKAA2, was found, further emphasizing the relevant role of AMPK in AD.

In superior frontal gyrus, the functional categories that are over-represented in significantly enriched reference modules converge not only to metabolic functions as in posterior cingulate cortex, but also to synaptic transmission. They comprise numerous neurotransmitter signaling pathways, including dopaminergic, GABAergic, glutamatergic, serotonergic, and neuropeptidergic systems (Figure 3). Altered cognition, learning and memory are clinical major features of AD and well known is the role of all major neurotransmitters in this higher brain function in physiological conditions and in AD [50]. Our findings give also support for a role of neuropeptidergic transmission in AD, in particular orexigenic neuropeptides (neuropeptide Y, orexin, agouti-related peptide, proopriomelnocortin, dynorphin, neuropeptide FF) that are involved in food intake and energy regulation. This advocates for a potential association to alteration of energy homeostasis in AD and AMPK, as this latter has been shown to mediate the orexigenic or anorexigenic effects of various neuropeptide signals [51]. AMPK appears also to couple energy metabolism to neuronal plasticity, as suggested by [52], thus linking energetic deficiency to alteration in synaptic transmission and memory impairment. This may possibly explain how memory could be controlled by energy metabolism, organization of the cytoskeleton and other biological processes relevant for neuronal survival.

The validity of the result were also tested using OMIM AD-related genes by adopting two strategies: OMIM genes were used (1) as a control, by checking their presence in reference modules found using the three original lists of *seed genes*, or (2) as a fourth list of *seed genes* and treated as an additional layer of evidence. In the first strategy, the significant enrichment of OMIM Alzheimer’s disease associated genes found in previously identified reference modules strengthens the conclusions of the three-level analysis. In the context of the second strategy, when used as an additional layer of evidence, the results did not perturb the findings for PC and SFG (Figure 2), thus confirming the robustness of our methodological approach to the addition of a new set of independent data.

Additionally, the presence of AMPK-related genes in this new set of reference modules passed two out of three of our significance tests. The additional enriched modules contributed by the OMIM *seed genes* list were biased in favor of well-known AD genes, and as a result AMPK-related genes did not reach significance threshold when tested for frequency in reference modules. Thus the negative outcome of the test simply reflects the fact that the addition of a list of known AD genes to the analysis has the effect of diluting the significance of newly discovered genes such as AMPK.

## Conclusions

In the present study, a novel multifactorial network analysis approach provided evidence, together with a number of recently published findings [53]–[55], suggesting that the deregulation of various metabolic factors and energy homeostasis, possibly determined by aging process, play a key role in AD. These processes possibly involve orexigenic neuropeptides and, particularly, AMPK. These alterations, in an adverse genetic environment, could explain the major hallmark of AD, tangle and plaques, all the modifications in metabolic signaling and cognitive functions, and the inflammatory and apoptotic events seen in AD. We hypothesize that these processes could be activated by the conflict between the low level of energy metabolism and the high level of regulatory and repair load, as suggested by Sun and colleagues [10]. Future studies will focus on the specific investigation of these metabolic alterations also on a systemic level, with the inclusion in the analysis of studies in blood samples from affected individual.

## Supporting Information

Figure S1 **Map of the gene expression signature of the samples from the GSE5281 dataset.** Each node represents a sample, and an edge between two nodes represents the distance between the respective transcriptional signatures. The spontaneous clustering of the samples in groups reflects the existence of classes of highly similar expression profiles corresponding almost perfectly to the tissue of origin (color legend: EC = orange, MTG = red, SFG = cyan, HIP = purple, VCX = yellow, PC = blue). The map was obtained with a signature size of 125+125 genes (up-regulated+down-regulated); only the top 20% of all pairwise distances were included in the map as edges.
(TIF)

Figure S2 **Schematic representation illustrating the procedure for identifying reference modules.** We use the giant component of HPRD to detect modules that are significantly enriched with respect to different lists of *seed genes*. Consider a hypothetical case with *seed genes* overrepresented in 10 modules, after running 100 times the “spinglass.community” algorithm. To understand possible redundancies (i.e., the fact that the same module is found during different runs), we have to compare size and composition of significantly enriched modules. First, enriched modules are classified on the basis of their size (e.g., in this hypothetical example, the 10 enriched modules are classified in four size categories: (a) 9, 9; (b) 80, 76 and 82; (c) 200, 480, 520 and 512; (d) 1023). If we focus on the first group, two enriched modules of size 9 are included. After classifying these two modules in the same size group, we have to determine whether they can be merged (i.e., they have to share some nodes and interactions so that *seed gene* enrichment found with constitutive modules is still significant after their union). At this step, there can be three possible scenarios: (1) the modules can be successfully merged, without altering the significance of the statistics (i.e., there are 4 *seed genes* out of 10 in the new reference module); (2) the modules share some nodes and connections, but after their merging the significant enrichment in *seed genes* vanishes (i.e., there are 3 *seed genes* out of 16, and the two initial modules should be considered as separate reference modules); (3) the two initial modules do not share any node (and connection), and have to be considered as representative of two different reference modules (where *seed genes* are significantly enriched with the following ratios: 3/9 and 4/9).
(EPS)

Table S1 **This file includes** ***seed genes*** **extracted from expression data of AD patients, SNPs that are significantly associated to AD, AD primary drug targets, and AD OMIM genes.** The remaining spreadsheets show the lists of genes composing enriched reference modules in AD-related data. They refer to reference modules detected using the differentially expressed genes, drug targets and OMIM genes alone (no enriched modules were found with SNPs), but also describe the composition of reference modules where genes from (1) the union of transcriptomic and genetic signatures, (2) the union of genes identified with transcriptomic, genetic and drug target, and (3) the union of transcriptomic, genetic, drug targets and OMIM genes data are over-represented.
(XLSX)

Table S2 **Summary of the specific GO terms corresponding to the main classes listed in** **Figures 2** **–** **3** **.**
(XLSX)

Tables S3 **Statistical analysis of the relatedness of AMPK signaling to AD.** (1) Frequency of AMPK nodes in the enriched reference modules, (2) Enrichment analysis specific to AMPK nodes and their direct neighbors, (3) Shortest distances linking AMPK nodes to *seed genes*.
(DOCX)
