# Supplementary material for: The Central Role of AMP-Kinase and Energy Homeostasis Impairment in Alzheimer’s Disease: A Multifactor Network Analysis
Source: PLoS One. 2013 Nov 12;8(11):e78919. doi: 10.1371/journal.pone.0078919 (PMC3827084; doi:10.1371/journal.pone.0078919)
Supplement: Tables S3 — Statistical analysis of the relatedness of AMPK signaling to AD. (1) Frequency of AMPK nodes in the enriched reference modules, (2) Enrichment analysis specific to AMPK nodes and their direct neighbors, (3) Shortest distances linking AMPK nodes to seed genes. (DOCX) [file pone.0078919.s005.docx]

**Supplementary Tables S5**

**More statistical analyses on the importance of AMPK**

Table SM5.1 summarizes the number of AMPK nodes that can be found in the lists of *seed genes*. Although the relevance of AMPK in AD has been partially described in literature [1,2], it is poorly documented by its recurrence in the lists of *seed genes*. Network analysis contributed to emphasize the importance of AMPK, suggesting this kinase as a potential key player in AD.

| ***Seed genes* (in HPRD)** | **AMPK nodes** |
| --- | --- |
| **Expression data** |  |
| EC (141) | 0 |
| HIP (126) | 1 |
| MTG (96) | 1 |
| PC (117) | 0 |
| SFG (110) | 1 |
| VCX (113) | 0 |
|  |  |
| **SNPs** |  |
| top10 (7) | 0 |
| whole (533) | 2 |
|  |  |
| **Drug targets (164)** | 2 |
|  |  |
| **OMIM (13)** | 0 |

**Table S5.1.** Overlap between *seed genes* at four layers of the model and the 9 AMPK nodes that can be found in the HPRD network. Next to each *seed gene* category we indicated, between parentheses, the actual number of genes in the HPRD network (e.g., 141 out of 250 differentially expressed genes specific to EC are in the HPRD network).

***(1) Frequency of AMPK nodes in the reference modules***

Frequency measures the number of times a node appears in enriched reference modules. To investigate whether the frequency of AMPK nodes was higher than the one of non-AMPK nodes, Wilcoxon rank sum test (equivalent to Mann-Whitney test) was applied. In case of p-values << 0.001, the frequency of AMPK-nodes is significantly higher than the one of non-AMPK nodes. We used this non-parametric test because (a) data are not independent (i.e., they are extracted from the same network) and (b) are not normally distributed (i.e., either all of the nodes showed the same frequency, or the Shapiro-Wilk test for normality was significant; p << 0.001). Table SM5.2 indicates p-values of the Wilcoxon rank sum test in case reference modules were identified using: (1) simple lists of *seed genes*; (2) union of expression data with SNPs; (3) union of expression data with SNPs and drug targets; (4) union of expression data with SNPs, drug targets and OMIM genes. We considered two scenarios, referring to the 9 AMPK nodes only, and also including their 25 direct neighbors.

- With the four levels of analysis, when using reference modules of specific brain regions (e.g., MTG modules with expression data only; SFG modules from the union of expression profiles and SNPs), the frequency of AMPK nodes in the reference modules was not significantly higher than the one of non-AMPK nodes.

- In case of the union of gene expression, SNPs and drug targets (i.e., when expression data for all brain regions were used), the frequency of AMPK nodes was higher than the one of the other nodes in reference modules. This is because AMPK nodes appeared many times in different enriched reference modules of PC and SFG brain regions.

| **Data type** | **p-value (9 AMPK)** | **p-value (9 AMPK + 25 neigh = 34)** |
| --- | --- | --- |
| **----1----** | | |
| Expression data | 0.078 | 0.180 |
| SNPs | no reference modules | |
| Drug targets | single reference module | |
| OMIM | no AMPK in reference modules | 0.940 |
| **----2----** | | |
| Expression data & SNPs | no AMPK in reference modules | 0.688 |
| **----3----** | | |
| **Expression data, SNPs & drug targets** | **<< 0.001** | **<< 0.001** |
| **----4----** | | |
| Expression data, SNPs, drug targets & OMIM | 0.769 | 0.890 |

**Table S5.2.** Data types and associated p-values from the Wilcoxon rank sum test. Significant p-values refer to cases for which AMPK nodes were found in reference modules with higher frequency than non-AMPK nodes. Significant scenarios (“Expression data, SNPs & drug targets”) are in bold. Expression data refers to the union of all expression profiles, while SNPs to the top 10 genes. Reference modules obtained from more specific data (i.e., EC, HIP, MTG, PC, SFG and VCX) never showed significant results. The two columns report results concerning the comparisons with the frequency of: 9 AMPK nodes only; 9 AMPK nodes together with their 25 direct neighbors (for a final subset of 34 nodes).

***(2) Enrichment analysis specific to AMPK nodes and their direct neighbors***

There are 9 AMPK nodes in the HPRD network, and they are characterized by 25 direct neighbors. From their union, a subset of 34 unique nodes was obtained. We tested whether different combinations of *seed genes* are over-represented in this group of 34 nodes. Results are shown in Table SM5.3; we distinguished between the short list of top 10 SNPs (7 of which are in HPRD) and the longer list of 2747 SNPs (533 are included in the HPRD network). Enrichment was estimated with hypergeometric test; adjusted p-values determined with the Benjamini & Hochberg correction [3].

- With the scenarios based on the top 10 SNPs, the sub-network composed of AMPK nodes and their direct neighbors was never enriched in presence of expression data, except in two cases: (a) the union of expression data with drug targets and OMIM genes; (b) the union of all *seed genes*.

- Scenarios based on the long list of SNPs are always characterized by significant enrichment of the 34 AMPK nodes with *seed genes*, with the exception of cases that rely on: (a) the whole expression data only; (b) the union of whole expression data with drug targets; (c) the union of whole expression data with OMIM genes.

- When using the long list of 533 SNPs, all combinations of *seed genes* that included the SNPs were significantly enriched in the sub-network composed of 9 AMPK nodes and their 25 direct neighbors.

|  | **hits (top 10 SNPs)** | **p-value** | **adjusted p-value** | **hits (whole SNPs)** | **p-value** | **adjusted p-value** |
| --- | --- | --- | --- | --- | --- | --- |
| **-----1-----** | | | | | | |
| E | 1 | 0.601 | 0.606 | 1 | 0.601 | 0.601 |
| **S** | **0** | **-** | **-** | **5** | **0.007** | **0.014** |
| **D** | **3** | **0.002** | **0.005** | **3** | **0.002** | **0.006** |
| **O** | **1** | **0.001** | **0.004** | **1** | **0.001** | **0.003** |
| **-----2-----** | | | | | | |
| **ES** | 1 | 0.606 | 0.606 | **6** | **0.057** | **0.071** |
| ED | 4 | 0.097 | 0.135 | 4 | 0.097 | 0.112 |
| EO | 2 | 0.332 | 0.388 | 2 | 0.332 | 0.355 |
| **SD** | **3** | **0.002** | **0.005** | **7** | **0.001** | **0.003** |
| **SO** | **1** | **0.002** | **0.005** | **5** | **0.007** | **0.014** |
| **DO** | **4** | **<< 0.001** | **0.002** | **4** | **<< 0.001** | **0.003** |
| **-----3-----** | | | | | | |
| **ESD** | 4 | 0.099 | 0.135 | **8** | **0.011** | **0.019** |
| **ESO** | 2 | 0.336 | 0.388 | **6** | **0.057** | **0.071** |
| **EDO** | **5** | **0.035** | **0.060** | **5** | **0.035** | **0.052** |
| **SDO** | **4** | **<< 0.001** | **0.002** | **7** | **0.001** | **0.003** |
| **-----4-----** | | | | | | |
| **ESDO** | **5** | **0.036** | **0.060** | **8** | **0.011** | **0.019** |

**Table S5.3.** Enrichment analysis in the sub-network composed of AMPK nodes and their direct neighbors (34 nodes). We tested whether *seed genes* that refer to differential expression (E), SNPs (S), drug targets (D) and OMIM (O) are significantly over represented in this small subset of 34 genes. Besides using simple gene lists (i.e., E, S, D, and O), we also tested their combinations. Grey shaded columns refer to the large SNPs group (533 nodes in HPRD), while white columns consider the 10 most significant SNPs (7 in HPRD). Significant results in bold (adjusted p-value threshold < 0.1).

***(3) Shortest distances linking AMPK nodes to seed genes***

Scope of this section was determining whether shortest paths linking the 34 AMPK nodes (they include the 9 nodes that are defined as AMPK, together with their direct neighbors) to *seed genes* were significantly shorter than the ones connecting the same *seed genes* to subsets of randomly sampled nodes from HPRD. Randomly sampled nodes did not include AMPK nodes and *seed genes*. For this study, we implemented the following procedure.

- We measured the shortest distances between the 34 AMPK nodes and the *seed genes* related to expression data (classified as the union of the 6 regions, or considering the 6 categories as different lists: EC, HIP, MTG, PC, SFG and VCX), SNPs (top 10 and the whole set of 533 SNPs), drug targets and OMIM genes. Data were collected as: (a) vectors of 34 elements summarizing average shortest paths linking each AMPK node to *seed genes* (“avg”); (b) lists of 34 vectors including shortest paths connecting each AMPK node to all *seed genes* (“all”).

- We extracted 1000 random vectors composed of 34 non-*seed genes* and non-AMPK nodes, measuring shortest distances to *seed genes* (both in terms of average - i.e., “avg” - and full distribution of shortest distances - i.e., “all” - as done with AMPK).

- The outputs obtained with the 1000 random vectors (i.e., average shortest distances, and the whole distributions of shortest distances to *seed genes*) were compared to results of AMPK nodes. We aimed at checking, with Wilcoxon signed rank test, whether AMPK nodes displayed shorter distances to *seed genes* than randomly chosen nodes. For each list of *seed genes*, this led to 1000 p-values in case of “avg” and 1000 p-values with “all” data.

- We combined the 1000 p-values found for each scenario into a unique p-value. We started from the fact that p-values should be uniformly distributed when the null hypothesis is true, and their cumulative distribution should approximate a normal distribution [4]. We used the (possible) deviation from the normal distribution to estimate whether the 1000 p-values of each set were lower than expected. Table SM5.4 summarizes the combined p-values, and indicates the number of p-values that are below the 0.05 threshold. Also if this number is small, the difference between AMPK and non-AMPK nodes can be significant (i.e., combined p-value < 0.05).

|  | **# p-values < 0.05 (“avg”)** | **p-value (“avg”)** | **# p-values < 0.05 (“all”)** | **p-value (“all”)** |
| --- | --- | --- | --- | --- |
| **Expression data** |  |  |  |  |
| All expression | 8 | 1.000 | 363 | 1.000 |
| EC | 4 | 1.000 | 318 | 1.000 |
| HIP | 6 | 1.000 | 303 | 1.000 |
| MTG | 9 | 0.921 | 363 | 0.531 |
| PC | 4 | 0.999 | 355 | 1.000 |
| SFG | 5 | 1.000 | 302 | 1.000 |
| VCX | 8 | 0.996 | 341 | 1.000 |
| **SNPs** |  |  |  |  |
| 10SNPs | 6 | 0.999 | 122 | 0.999 |
| **All SNPs** | **8** | **<< 0.001** | **516** | **<< 0.001** |
| **Drug targets** | **9** | **<< 0.001** | **449** | **<< 0.001** |
| **OMIM** | **5** | **0.049** | **217** | **0.014** |

**Table** **S5.4.** Combined p-values summarizing the results of Wilcoxon signed rank tests. These tests were used to compare the differences in the shortest distances separating AMPK and non-AMPK (also non-*seed gene*) nodes to *seed genes*. Distances to *seed genes* were estimated by the distributions of average shortest paths (“avg”) and their global patterns (“all”). AMPK nodes showed significantly shorter distances to SNPs (533 genes), drug targets and OMIM nodes, if compared to random lists of non-AMPK and non-*seed genes*; significant results in bold (p-value threshold < 0.05).

**References**

1. Cai Z, Yan LJ, Li K, Quazi SH, Zhao B (2012) Roles of AMP-activated Protein Kinase in Alzheimer’s Disease. NeuroMolecular Medicine 14: 1-14.
2. Salminen A, Kaarniranta K, Haapasalo A, Soininen H, Hiltunen, M (2011) AMP‐activated protein kinase: a potential player in Alzheimer’s disease. Journal of Neurochemistry 118: 460-474.
3. Benjamini Y, Hochberg Y (1995) Controlling the false discovery rate: a practical and powerful approach to multiple testing. Journal of the Royal Statistical Society Series B 57: 289-300.
4. Murdoch D, Tsai Y, Adcock J (2008) P-Values are Random Variables. The American Statistician 62: 242-245.
